# Supplementary material for: Transcriptomic and Metabolomic Responses in Cotton Plant to Apolygus lucorum Infestation
Source: Insects. 2022 Apr 15;13(4):391. doi: 10.3390/insects13040391 (PMC9025427; doi:10.3390/insects13040391)
Supplement: Supplementary file 1 [file insects-13-00391-s001.zip › Figure S1.pdf]

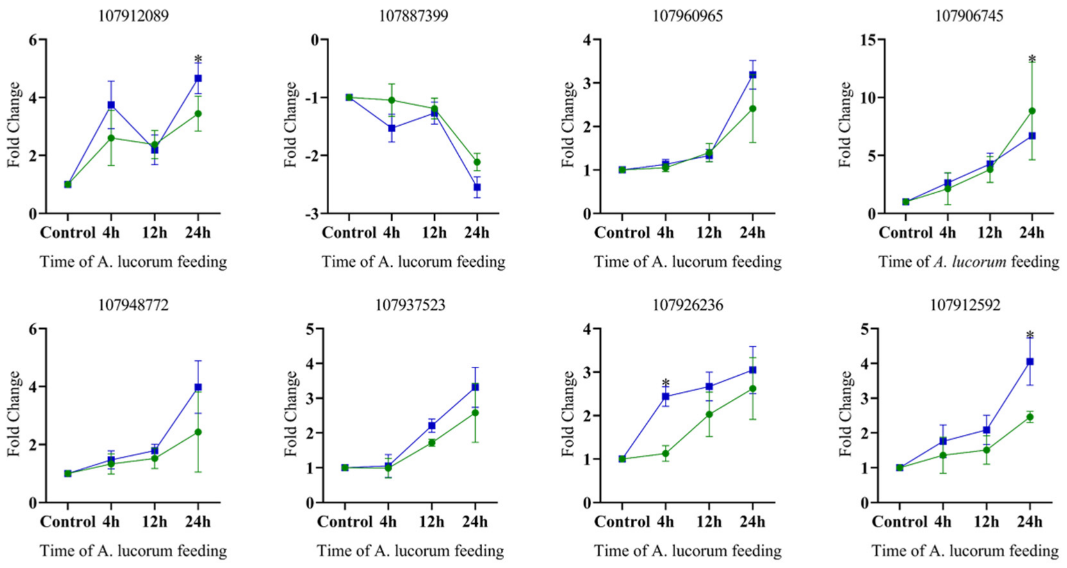

Figure S1. Comparison of expression levels of selected genes in RNA-seq and qRT-PCR analysis.

Asterisks indicate statistically significant differences compared to control samples. * $p< 0.05$, (Student's t test)
